# ADME Gene-Related Pharmacogenomic Labeling of FDA-Approved Drugs: Comparison with Clinical Pharmacogenetics Implementation Consortium (CPIC) Evidence Levels

**DOI:** 10.3390/medicines11030006

**Published:** 2024-02-20

**Authors:** Subrata Deb, Robert Hopefl, Anthony Allen Reeves, Dena Cvetkovic

**Affiliations:** Department of Pharmaceutical Sciences, College of Pharmacy, Larkin University, Miami, FL 33169, USA

**Keywords:** pharmacogenomics, FDA, drug label, Clinical Pharmacogenetics Implementation Consortium, cytochrome P450, phase I, phase II, transporter, ADME

## Abstract

Pharmacogenomics (PGx) can facilitate the transition to patient-specific drug regimens and thus improve their efficacy and reduce toxicity. The aim of this study was to evaluate the overlap of PGx classification for drug absorption, distribution, metabolism, and elimination (ADME)-related genes in the U.S. Food and Drug Administration (FDA) PGx labeling and in the Clinical Pharmacogenetics Implementation Consortium (CPIC) database. FDA-approved drugs and PGx labeling for ADME genes were identified in the CPIC database. Drugs were filtered by their association with ADME (pharmacokinetics)-related genes, PGx FDA labeling class, and CPIC evidence level. FDA PGx labeling was classified as either actionable, informative, testing recommended, or testing required, and varying CPIC evidence levels as either A, B, C, or D. From a total of 442 ADME and non-ADME gene–drug pairs in the CPIC database, 273, 55, and 48 pairs were excluded for lack of FDA labeling, mixed CPIC evidence level provisional classification, and non-ADME gene–drug pairs, respectively. The 66 ADME gene–drug pairs were classified into the following categories: 10 (15%) informative, 49 (74%) actionable, 6 (9%) testing recommended, and 1 (2%) testing required. CYP2D6 was the most prevalent gene among the FDA PGx labeling. From the ADME gene–drug pairs with both FDA and CPIC PGx classification, the majority of the drugs were for depression, cancer, and pain medications. The ADME gene–drug pairs with FDA PGx labeling considerably overlap with CPIC classification; however, a large number of ADME gene–drug pairs have only CPIC evidence levels but not FDA classification. PGx actionable labeling was the most common classification, with CYP2D6 as the most prevalent ADME gene in the FDA PGx labeling. Health professionals can impact therapeutic outcomes via pharmacogenetic interventions by analyzing and reconciling the FDA labels and CPIC database.

## 1. Introduction 

Pharmacogenomics (PGx) is the study of genetic differences that influence the variability in drug response [1]. PGx is an important clinical factor to consider when maximizing patient treatment outcomes and reducing adverse events by providing individualized care. PGx knowledge can facilitate the transition to patient-specific drug regimens, thus improving efficacy and reducing toxicity [1]. The major components of pharmacokinetics (PK), such as absorption, distribution, metabolism, and elimination (ADME), can be affected by PGx. Absorption is the crossing of a drug from administration to the systemic circulation, while distribution is perfusing different organs with drug-containing plasma [2]. Biotransformation or metabolism is the process of enzymatic modification of the drug to typically make it more water-soluble, usually preceding the elimination of the drug from the body [2]. Polymorphism refers to the presence of two or more variants of a gene that can occur in different individuals or in different populations [3]. Genes related to the drug ADME or PK properties are susceptible to genetic polymorphisms and may influence the pharmacokinetic behavior of medications [2]. Polymorphisms can affect drug absorption or drug metabolism via variations in transporters and cytochrome P450 (CYP) enzymes, respectively, and can result in altered therapeutic outcomes or adverse effects [4]. The knowledge of PGx affords an overall decrease in the cost of health care, optimizing outcomes of patient therapy, and improved medication alternatives [5]. 

Enzymes (e.g., CYP) involved in phase I metabolism modify drug structure via oxidation, reduction, hydrolysis, cyclization, or by the deletion of hydrogen or incorporation of oxygen [6]. Phase II metabolism reactions are catalyzed by enzymes such as uridine 5′-diphospho glucuronosyltransferase 1A1 (UGT1A1) and thiopurine S-methyltransferase (TPMT) and involve combining a phase I metabolite with an endogenous molecule via conjugation, generally resulting in an inert and more hydrophilic compound [6]. Transporters, e.g., solute carrier organic anion transporter family member 1B1 (*SLCO1B1*), are proteins that transport compounds from the gastrointestinal lumen to systemic circulation and to the interior of the cell [7]. Phase I/II enzymes and transporters are the major contributors to drug PK properties and are susceptible to polymorphic forms. A common example of PGx-related drug toxicity from phase I enzymes can be illustrated via the relationship between warfarin and *CYP2C9*, where individuals with *2 and *3 variants of *CYP2C9* are known to be associated with increased anticoagulation effects and bleeding [8]. PGx can also cause subtherapeutic response to a drug, such as the case with clopidogrel (antiplatelet) and *CYP2C19*, where carriers of no function allele have significantly reduced clopidogrel active metabolite formation leading to serious cardiovascular events [9]. 

Awareness about drug-related PGx information among researchers, healthcare professionals, and regulatory agencies contributes to optimum PGx benefits. The Pharmacogenomics Knowledge Base (PharmGKB), which was sponsored by the National Institutes of Health (NIH) and the National Institute of General Medical Sciences (NIGMS), was established in 2000 [10]. It houses PGx data on different variants published in original research papers and provides pharmacokinetic and pharmacodynamic pathways of pertinent drugs [11]. Subsequently, the Clinical Pharmacogenetics Implementation Consortium (CPIC), a combined effort by PharmGKB and Pharmacogenomics Research Network (PGRN), was created in 2009 in response to the needs identified for practice guidelines and the intent to apply the available PGx information in practice [12]. The CPIC curates evidence and assigns a level of A, B, C, and/or D. Each level corresponds to the amount of clinical evidence available, with level A denoting the highest level of evidence [13]. Based on the present PGx evidence, specific gene–drug pairs are identified as single or mixed levels (e.g., A/B, B/C, C/D) and are subject to change as additional evidence is reported. The United States Food and Drug Administration (FDA) has recognized certain drugs that are susceptible to genetic influences by including PGx information in the drug labels or package inserts and web resources [14]. Based on the evidence provided by the drug developer during approval and post-marketing research, the FDA has developed a table of certain drugs and their PGx biomarkers in drug labeling. The PGx-related information on package inserts may contain details on genetic variants that can affect plasma drug concentration and drug action, the potential to experience toxicity, variant-targeted drug administration, and other clinical characteristics [14]. According to the PharmGKB annotations, the FDA PGx labeling can be interpreted as either actionable, informative, testing recommended, or testing required [13]. The CPIC guidelines help healthcare personnel utilize PGx knowledge from a clinical and scientific perspective, whereas FDA labeling info facilitates the implementation from a regulatory and patient safety perspective. However, due to the differences in categorization terminologies and implementation strategies, as such, there is no effort to consolidate these resources, which could pose real-life challenges in their routine utilization. The limited availability of literature combined with the lack of consistency in clinical guidelines and the extent of intervention needed appear to impact the implementation of PGx evidence [12,15,16]. 

For the purpose of this work, the genes related to PK properties are labeled as “ADME genes”, whereas genes that are related to pharmacodynamic properties of the drug (mechanism of action) are termed as “non-ADME genes”. The aim of this study was to evaluate the overlap of PGx categorization for ADME gene–drug pairs between FDA PGx labeling classifications (actionable, informative, testing required, and testing recommended) and the CPIC evidence-level classifications (A, B, C, or D). Gene–drug pairs with only one CPIC evidence level were included in the study, as pairs with mixed provisional levels (e.g., A/B, C/D) are considered to have inconclusive evidence. A secondary objective was to determine the prevalence of ADME genes common between FDA PGx labeling and the CPIC database. Although the PGx information stems from a particular gene and drug molecule and is not disease-specific, the distribution of the PGx-relevant drugs used to treat different disease conditions was also explored in the present study. This will give perspective in terms of the utility of PGx information in the treatment outcomes of certain disease conditions and draw the attention of healthcare professionals. Overall, the present work is relevant to highlight the strength of evidence for certain ADME gene–drug pairs present in both FDA and CPIC databases and also indicates that additional reconciliation efforts are needed to bridge the gap between the two major PGx databases.

## 2. Materials and Methods

### 2.1. Data Sources/Collection

The CPIC database (https://cpicpgx.org, accessed on 5 July 2021) was searched for FDA-approved drugs with PGx labeling (https://www.fda.gov/drugs/science-and-research-drugs/table-pharmacogenomic-biomarkers-drug-labeling, accessed on 5 July 2021) for inclusion in the initial analysis. The CPIC database provides a comprehensive list of gene–drug pairs and corresponding guidelines to clinically implement pharmacogenetic information [13]. The initial collection of gene–drug pairs consisted of all FDA-approved drugs with PGx labeling, regardless of relation to ADME (PK-related) or non-ADME genes. A non-ADME (pharmacodynamic-related) gene–drug pair involves a gene that does not influence drug ADME but affects other components of drug action.

### 2.2. Data Characteristics and Annotations

The CPIC database pairs drugs with their related genes, either ADME (PK-related) or non-ADME (pharmacodynamic-related). Gene–drug pairs were filtered by their association with ADME (PK)-related genes, FDA PGx labeling, and CPIC classification. ADME-related genes were defined as the proteins involved in phase I/II metabolism or drug transport. PharmGKB annotates the FDA drug labels in the PGx levels of either actionable, informative, testing required, or testing recommended [13]. Actionable PGx means that the package insert may have facts about altered drug action and dose as a result of genetic variants and metabolizer status. The label may contain contraindications of a medication in a section of individuals with a specific genetic configuration and functionality without mandatory genetic testing [10]. Informative PGx means the package insert has gene-related details that affect the dosage, metabolism, or toxicity, which are not clinically significant. Drugs are also placed in this category if they do not meet the criteria to place them as testing required, testing recommended, or actionable [10]. Testing required suggests genetic testing for the gene(s) related to pharmacokinetics or pharmacodynamics must be carried out before drug administration to avoid life-threatening drug action outcomes. Testing recommended means that the genetic information in the package insert is highly encouraged, but it is not mandatory to conduct genetic testing before using this drug [10]. Likewise, based on the strengths of the evidence available, CPIC classifies the gene–drug pair PGx as A, B, C, or D. CPIC evidence level A means a strong or moderate PGx intervention is recommended, whereas level B means a discretionary PGx action is recommended. For level C or level D, no prescription changes are required due to either variable or very little PGx evidence available at this time [13]. The intervention in the therapeutic strategy is driven by the nature of the evidence available. 

### 2.3. Data Analysis

Data were analyzed via descriptive statistics and percentages. Data were downloaded from the CPIC database into Microsoft Excel prior to analysis. All flow charts and graphs were created using Microsoft Excel and Microsoft Word.

#### 2.3.1. ADME Gene and Drug Pairs with PGx FDA Labeling

In the first analysis, PGx levels of each ADME or non-ADME gene–drug pair in the CPIC database were included. Subsequently, gene–drug pairs without PGx FDA labeling and gene–drug pairs with mixed CPIC evidence levels of A/B, B/C, and C/D were excluded in a stepwise manner. Non-ADME-drug and ADME gene–drug pairs were included initially, followed by the exclusion of all non-ADME genes, as the focus of the current work was to understand the ADME gene-related PGx evidence.

#### 2.3.2. CPIC Evidence Levels of ADME Gene–Drug Pairs with FDA Labeling Classification 

An analysis was conducted to determine the prevalence of different CPIC evidence levels for ADME gene–drug pairs in FDA labeling classifications. The gene–drug pairs with informative FDA labeling were evaluated for CPIC evidence level A, B, C, or D. Similarly, the gene–drug pairs with either actionable, testing recommended, or testing required FDA labeling categories were analyzed for different CPIC level evidence. 

#### 2.3.3. ADME Gene Categories in FDA Labeling Classification

The prevalence of genes involved in different components of ADME, such as phase I enzymes, phase II enzymes, and transporters in each FDA labeling classification, was analyzed. Examples of phase I genes include *CYP2C9*, *CYP2C19*, *CYP2D6*, *CYP2B6*, and *DPYD*, whereas examples of phase II genes include *UGT1A1* and *TPMT*. *SLCO1B1* was the only transporter with both FDA PGx labeling and CPIC evidence level analyzed in the current study. Analysis of the CPIC data was carried out from both phase I, phase II, and transporters classification, as well as from the individual gene perspective. First, the ADME gene–drug pairs with informative FDA labeling and a CPIC level were evaluated for their presence either in phase I, phase II, or transporter categories. Likewise, the gene–drug pairs with either actionable, testing recommended, or testing required FDA labeling and a CPIC level were analyzed for categorizing them as phase I, phase II, or transporter related. Subsequently, the same process was repeated to identify the individual phase I, phase II, or transporter genes among the ADME gene–drug pairs with either informative, actionable, testing recommended, or testing required FDA labeling and one of the CPIC level categorizations. 

## 3. Results

### 3.1. Disease Spectrum of ADME Gene–Drug Pairs with CPIC and FDA PGx Labeling 

The initial examination of the ADME gene–drug pairs with a PGx FDA labeling and an assigned CPIC evidence level showed that they are unequally distributed across a variety of disease states (Table 1). The most prevalent disease state was depression (14 gene–drug pairs), followed by cancer (9 gene–drug pairs), gastroesophageal reflux disorder (6 gene–drug pairs), and pain (6 gene–drug pairs) (Table 1). *CYP2C19*-related drugs were used in diverse disorders, including depression, gastroesophageal reflux disorder, epilepsy, acute coronary syndrome, and stroke. *CYP2D6* gene had the largest number of drugs affected by its polymorphism forms with disease conditions such as depression, psychosis, pain, and epilepsy. Anticoagulants, antiepileptics, and pain drugs are examples of *CYP2C9*-related PGx labeling. Interestingly, both the phase II genes (*UGT1A1* and *TPMT*) predominantly affect drugs for cancer. The only transporter that has both FDA and CPIC classification affects statin drugs.

### 3.2. ADME Gene–Drug Pairs with Both PGx FDA Labeling and CPIC Categorizations 

**Figure 1:**
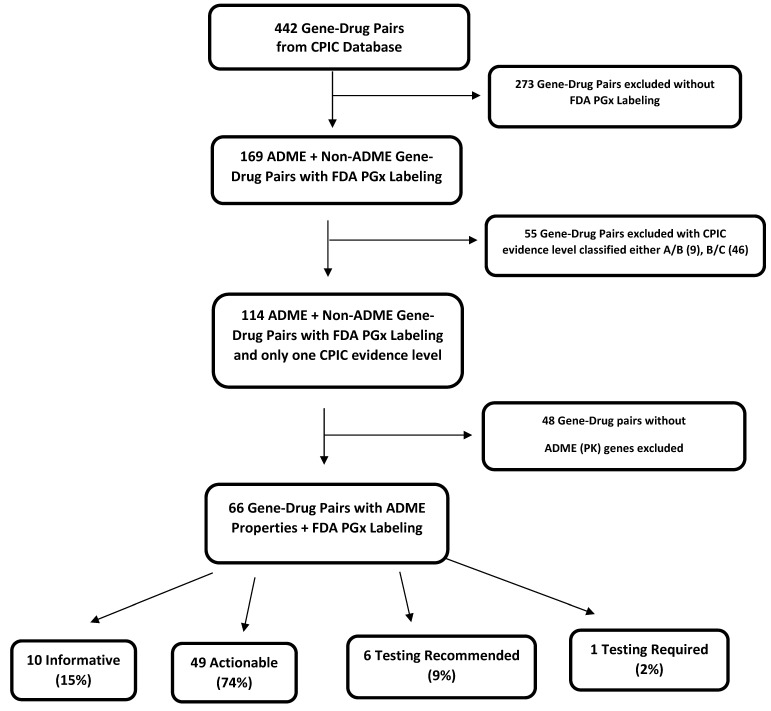
Flowchart depicting ADME gene–drug pairs with FDA PGx labeling found in CPIC database. Mixed provisional CPIC evidence levels (A/B or B/C) were excluded due to inconclusive evidence.

A total of 442 gene–drug pairs involving both non-ADME and ADME genes were included at the beginning of the analysis (Figure 1). From that pool, 273 gene–drug pairs were excluded that did not have any PGx FDA labeling, followed by the exclusion of 55 gene–drug pairs with mixed provisional CPIC evidence levels assigned as A/B or B/C. Since gene–drug pairs with mixed provisional CPIC evidence level classification did not allow us to include them in one or the other CPIC categories (A, B, C, or D) for quantitative analyses, they were excluded. At that stage, 114 non-ADME and ADME gene–drug pairs with FDA PGx labeling and only one CPIC evidence level remained with the following distribution: informative: 11 (10%), actionable: 86 (75%), testing recommended: 8 (7%), and testing required: 9 (8%). Further analysis was conducted only with ADME genes. From the pool of 114 non-ADME and ADME genes, 48 pairs without ADME properties were excluded as the current study solely focused on the genes (phase I/phase II enzymes and transporters) that are involved in pharmacokinetics (Figure 1). This resulted in 66 gene–drug pairs with ADME properties and FDA PGx labeling in the following labeling classes: informative: 10 (15%), actionable: 49 (74%), testing recommended: 6 (9%), and testing required: 1 (2%) (Figure 1 and Table 2). Table 2 lists each ADME gene–drug pair classified by FDA PGx labeling (informative, actionable, testing recommended, and testing required) and their corresponding CPIC evidence levels A, B, C, or D for each FDA class. 

### 3.3. Distribution of CPIC Evidence Levels in FDA Classifications of ADME Gene–Drug Pairs 

**Figure 2:**
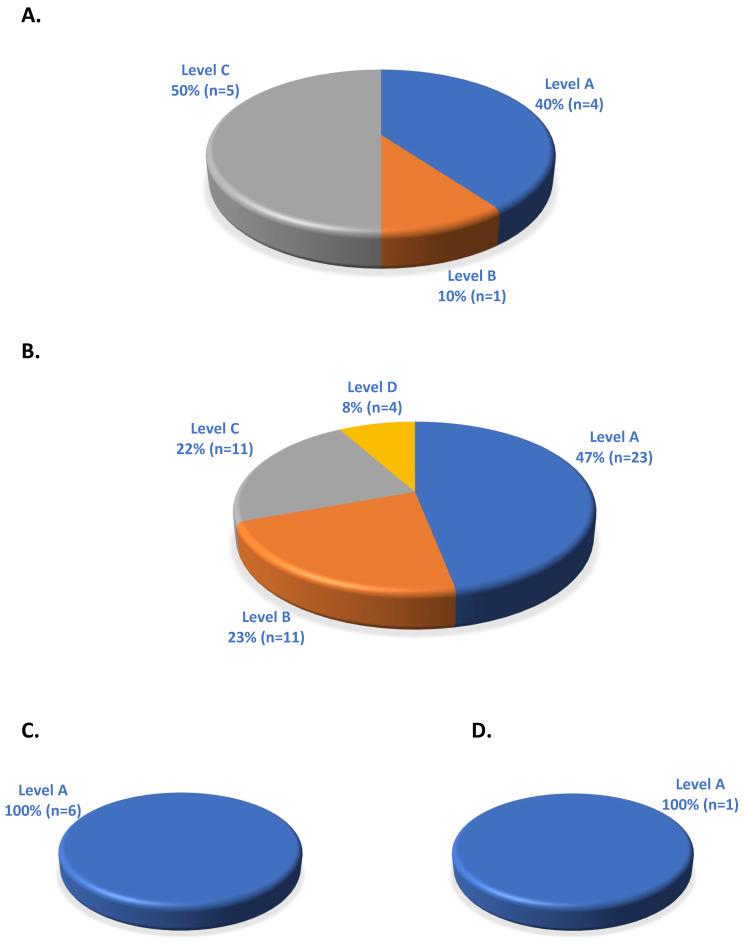
ADME gene–drug pairs with both FDA PGx labeling and CPIC evidence-level classification. (**A**) Informative (total 10). (**B**) Actionable (total 49). (**C**) Testing recommended (total 6). (**D**) Testing required (total 1).

There were 10 gene–drug pairs with PGx informative FDA labeling with CPIC evidence level A: 4 (40%), CPIC evidence level B: 1 (10%), CPIC evidence level C: 5 (50%), and CPIC evidence level D: 0 (0%) (Figure 2A). Similarly, for PGx FDA-actionable labeling, there were 49 gene–drug pairs with CPIC evidence level A: 23 (47%), CPIC evidence level B: 11 (23%), CPIC evidence level C: 11 (22%), and CPIC evidence level D: 4 (8%) (Figure 2B). In the analysis of PGx testing recommended FDA labeling, all six gene–drug pairs were from CPIC evidence level A classification (100%) (Figure 2C). In the PGx testing required FDA labeling category, only one gene–drug pair was found, and that was with CPIC evidence level A classification (100%) (Figure 2D). 

### 3.4. Prevalence of ADME Genes in PGx FDA Classifications

**Figure 3:**
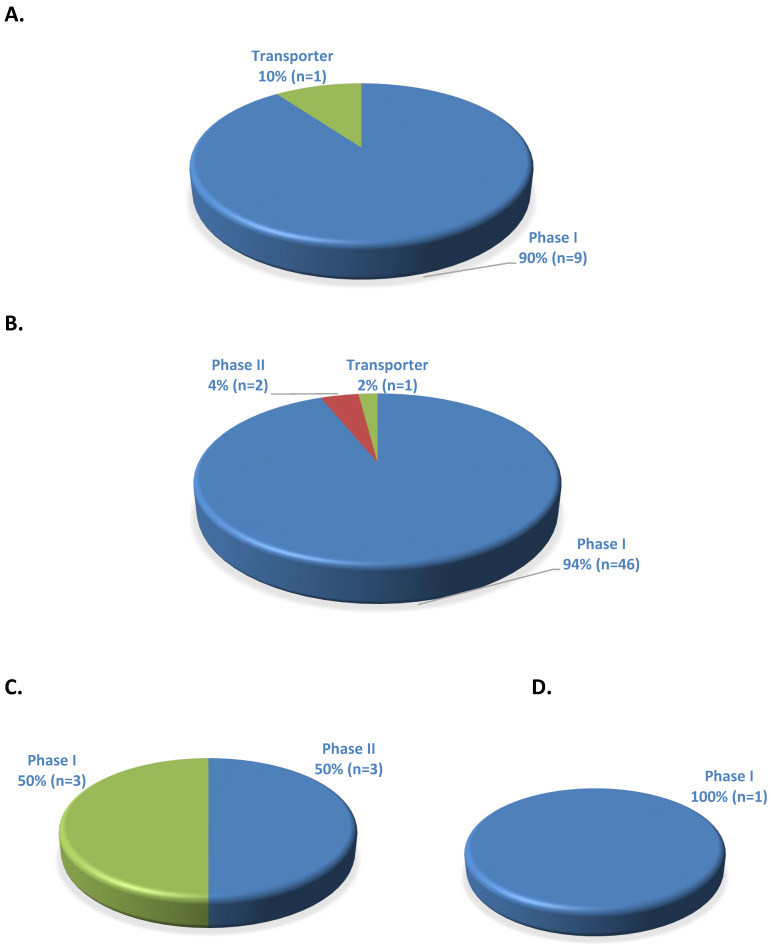
ADME gene–drug pairs with FDA PGx labeling in phase I, phase II, or transporter categories found in the CPIC database. (**A**) Informative (total 10), (**B**) Actionable (total 49), (**C**) Testing recommended (total 6), (**D**) Testing required (total 1).

**Figure 4:**
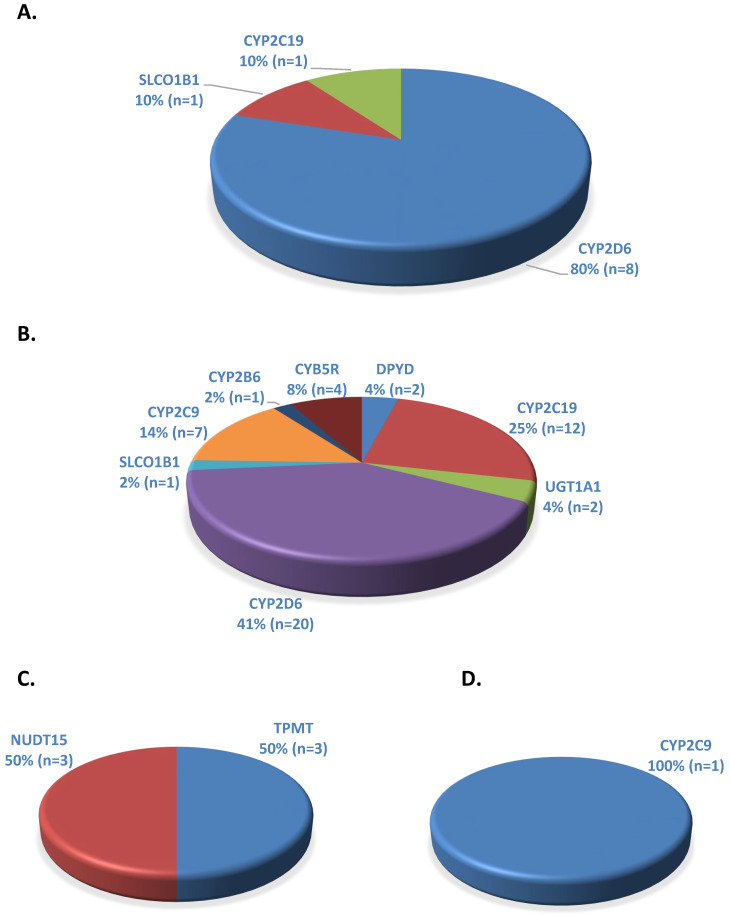
Individual ADME gene–drug pairs with FDA PGx labeling found in the CPIC database: (**A**) Informative (total 10), (**B**) Actionable (total 49), (**C**) Testing recommended (total 6), (**D**) Testing required (total 1).

Of the 10 ADME gene–drug pairs with PGx informative FDA labeling and a CPIC level, nine (90%) were related to phase I enzymes and one (10%) to transporter (Figure 3A). The individual ADME genes with PGx informative FDA labeling include *CYP2D6*: 8 (80%), *CYP2C19*: 1 (10%), and *SLCO1B1*: 1 (10%) (Figure 4A). In contrast, there were 49 ADME gene–drug pairs with PGx actionable FDA labeling and a CPIC level under phase I: 46 (94%), phase II: 2 (4%), and transporter: 1 (2%) categories (Figure 3B). Seven different genes were identified in the PGx actionable FDA labeling category with the following distribution: *CYP2D6*: 20 (41%), *CYP2C19*: 12 (25%), *CYP2C9*: 7 (14%), *DPYD*: 2 (4%), *CYB5R*: 4 (8%). *UGT1A4*: 2 (4%), *SLCO1B1*: 1 (2%), and *CYP2B6*: 1 (2%) (Figure 4B). For the ADME gene–drug pairs with PGx testing recommended FDA labeling and a CPIC level, six pairs were related to phase I: 3 (50%) and phase II: 3 (50%) enzymes (0%) (Figure 3C). *NUDT15* and *TPMT* were the phase I and phase II enzymes, respectively, in this category. In the analysis of ADME gene–drug pairs with PGx testing required FDA labeling and a CPIC level, there was only one pair with *CYP2C9* phase I enzyme (Figure 3D and Figure 4D). 

## 4. Discussion

PGx presents a unique opportunity to transition to a personalized medicine approach in which optimal drug choices and doses can be used to improve patient treatment outcomes [1]. The FDA recognizes that certain drugs can be impacted by genetic polymorphisms in proteins involved in drug metabolism and transport, thus influencing their ADME (pharmacokinetic) profile [14]. Awareness among healthcare providers about PGx remains limited [16,17,18]. The purpose of this study was to analyze different FDA PGx classifications for ADME gene–drug pairs in the CPIC database. 

While determining the overlap of PGx information in FDA labels and CPIC classification for gene–drug pairs, it was evident that the CPIC database contains additional PGx information compared to FDA PGx labels and medications for certain diseases more commonly have PGx information. It is pertinent to highlight that PGx is related to the gene and drug molecule; however, the condition(s) in which a particular drug is used is also of interest as the therapeutic outcomes will be affected due to the PGx effect and should draw the attention of the healthcare professionals. Depression, cancer, gastroesophageal reflux disorder, and pain medications are related to the majority of the ADME gene–drug pairs with both PGx FDA labeling and a CPIC level. Actionable FDA classification was by far the most prevalent in the ADME gene–drug pairs analyzed in this study, which implies that there is significant scope for pharmacogenetic intervention by healthcare providers. Interestingly, PGx actionable FDA labeling drugs are largely comprised of CPIC evidence level A, which suggests that the actionable gene–drug pairs have a high level of PGx evidence. Phase I enzymes constitute about most of the PGx actionable FDA labeling with *CYP2D6, CYP2C19,* and *CYP2C9* being the most commonly found ADME genes in that category. Similarly, *CYP2D6* was the most prevalent in the PGx informative FDA labeling. In the PGx informative FDA labeling, the gene–drug pairs were with CPIC level A or level C and thus infer that PGx evidence strength needs to be evaluated on a drug-by-drug basis. *CYP2C9*, a phase I enzyme, was the only gene with PGx testing required FDA labeling. For PGx testing recommended and testing required FDA labeling, all the gene–drug pairs had CPIC evidence level A, suggesting that either dose adjustment or a change in prescription is needed for those drugs. 

*CYP2D6* was the most prevalent gene with drugs having actionable FDA classification due to several polymorphic forms of *CYP2D6* and its involvement in the metabolism of diverse classes of medications. It was estimated that the probability of an individual having an altered metabolizer status (poor, intermediate, or ultrarapid metabolizer) is approximately 36.4% worldwide, with significant variations of frequency between different countries [19]. *CYP2D6* and codeine is a pharmacogenetically well-characterized gene–drug pair with FDA-actionable classification in which pharmacogenomic interventions can be utilized. Codeine is a prodrug that is O-demethylated into morphine (active drug) via *CYP2D6* [20]. In poor metabolizers, there may be subtherapeutic levels of morphine leading to uncontrolled pain, while ultrarapid metabolizers may experience adverse effects due to supratherapeutic levels [21,22]. Patient populations with severe and/or chronic pain can significantly benefit from *CYP2D6* PGx-guided therapy. A recent clinical trial utilized *CYP2D6* genotyping to guide pain management and concluded that *CYP2D6*-guided therapy showed greater improvement versus traditional care in a composite pain outcome for intermediate and poor metabolizers [23]. Optimum dosing for adequate pain control is imperative for patients taking opioids because of the significant abuse potential. In the case of *CYP2D6* ultrarapid metabolizers, more accurate dosages could be prescribed to mitigate some of the abuse potential of codeine. Similarly, other drugs, especially those with narrow therapeutic index, metabolized by *CYP2D6* (e.g., antiarrhythmics such as flecainide) and *CYP2C9* (e.g., anticoagulants such as warfarin) also present an opportunity to make pharmacogenomic interventions to improve patient care. 

Since, in the current study, depression is the disease state related to the greatest number of PGx gene–drug pairs having both FDA/CPIC categorizations, it is worth highlighting that *CYP2C19* and *CYP2D6* are primarily responsible for the metabolism of those antidepressant medications. For example, amitriptyline, a commonly used tricyclic antidepressant, is converted to nortriptyline (an active metabolite and an approved antidepressant) by *CYP2C19*, whereas *CYP2D6* catalyzes the formation of less active and cardiotoxic metabolites known as 10-hydroxy amitriptyline [24]. *CYP2D6*-amitriptyline and *CYP2C19*-amitriptyline gene–drug pairs have CPIC level A classification; however, only the *CYP2D6*-amitriptyline pair has both PGx actionable FDA labeling and CPIC level A categorization. This is a classic example of the gap between CPIC and FDA PGx labeling. In spite of the established CPIC guidelines [25], the FDA package insert does not have any PGx labeling for *CYP2C19*-amitriptyline. This pharmacogenetically well-studied drug has the potential to demonstrate a lack of efficacy in *CYP2D6* ultrarapid metabolizers or cause higher side effects in *CYP2D6* poor/intermediate metabolizers. For ultrarapid or poor metabolizers, a non-*CYP2D6* metabolized drug is recommended, whereas for intermediate metabolizers lowering of 25% dose is advised [25]. In contrast, *CYP2C19*-amitriptyline CPIC PGx guidelines provide information on the risk of higher formation of active metabolites from amitriptyline in *CYP2C19* ultrarapid metabolizers or lower efficacy in *CYP2C19* poor metabolizers due to lack of formation of nortriptyline (active metabolite) [25]. Thus, for amitriptyline, healthcare providers need to refer to both the CPIC database and FDA labeling to implement maximum PGx benefits for the patients. 

CPIC database has emerged as one of the most complete and applied resources for PGx information due to the comprehensive literature evaluations and inclusion of drugs with and without PGx labeling from the FDA [13]. Although there is an overlap between FDA PGx labeling and CPIC categorization level, there is still a significant amount of PGx information missing from FDA-approved drug labels when compared to the CPIC database. Analyses from other researchers have come to similar conclusions about the lack of adequate PGx information in FDA drug labeling [26,27,28]. The gap between FDA and CPIC PGx categorization has potential clinical applications as healthcare professionals may experience uncertainties in their efforts to apply the PGx guidelines. It is important to highlight that FDA and CPIC use two different rating systems, e.g., CPIC evidence level A does not automatically mean “testing required”, which makes it harder to convert the PGx information at the practice level. Since FDA package inserts and databases are likely most commonly used by healthcare professionals compared to other regulatory or clinical databases, the limited overlap between CPIC and FDA can potentially affect the ability of healthcare teams to implement available PGx knowledge in application. It can be inferred that the presence of certain ADME gene–drug pairs in both FDA and CPIC databases strengthens their evidence; however, additional reconciliation efforts are needed to include all CPIC gene–drug levels in the FDA PGX labeling. FDA PGx actionable labeling was the most prevalent labeling in the current study and represents an opportunity to make clinical recommendations to improve patient care. Actionable classification is the only FDA PGx labeling that contains information about changes in efficacy, dose, or metabolism that a health professional can consider while making dosage adjustments or other recommendations [10,14]. This contrasts with the other FDA PGx classifications (testing required, testing recommended, and informative), which leaves relatively less opportunity for a health professional to apply their clinical judgment. In addition, the availability of resources to conduct genetic testing could be another challenge in the implementation of FDA drug label guidelines. Pharmacists are medication experts with the knowledge of clinical pharmacology, including the ability to interpret genetic test results, and thus can intervene in minimizing toxicities and maximizing therapeutic benefits from FDA-approved drugs. However, the lack of consistency in categorization between two major PGx governing bodies in the United States may hinder the adoption of PGx in practice and add to existing challenges of PGx implementation in routine use. Understanding of PGx labeling for FDA-approved gene–drug pairs in comparison to the CPIC database will likely highlight the need to refer to appropriate PGx resources and generate awareness about the potential application of PGx among health professionals and improve patient treatment outcomes.

## 5. Conclusions

This study showed that there is a considerable overlap (58%) between FDA PGx labeling and CPIC categorization for ADME gene–drug pairs; however, a large number of ADME gene–drug pairs only have CPIC evidence-level categorization but not FDA classification. The most prevalent FDA PGx classification was actionable, with the most prevalent gene being *CYP2D6*. The majority of the genes with FDA PGx labeling and CPIC categorization were related to phase I metabolism. Medications indicated for depression constitute the largest number of drugs with FDA PGx labeling in the CPIC database. Health professionals can make significant dose adjustments and interventions via pharmacogenomic considerations, particularly in drugs with FDA PGx actionable labeling. The CPIC database and FDA PGx labeling should be reconciled by health professionals to provide optimal drug therapy while ensuring patient safety.

## Figures and Tables

**Table 1:** ADME gene–drug pairs with both FDA PGx labeling and CPIC classifications and their related disease states/drug class.

ADME Process	Gene	Examples of Drugs	Disease State/Drug Class
Phase I enzymes			
	CYP2C19	Citalopram, Escitalopram, Doxepin	Depression
		Clopidogrel	Antiplatelet
		Omeprazole, Lansoprazole, Pantoprazole, Esomeprazole, Dexlansoprazole, Rabeprazole	Gastroesophageal reflux disorder
		Voriconazole	Antifungal
		Brivaracetam	Antiepileptic
	CYP2D6	Amitriptyline, Nortriptyline, Paroxetine, Clomipramine Desipramine, Doxepin Fluvoxamine, Imipramine, Trimipramine, Duloxetine, Fluoxetine	Depression
		Atomoxetine	ADHD
		Codeine, Tramadol	Pain
		Ondansetron, Palonosetron	Nausea
		Pitolisant, Modafinil	Narcolepsy
		Tamoxifen	Breast cancer
		Aripiprazole, Risperidone	Antipsychotic
		Darifenacin, Fesoterodine, Tolterodine	Urinary incontinence
	CYP2C9	Celecoxib, Flurbiprofen, Meloxicam, Piroxicam	Pain
		Phenytoin, Fosphenytoin	Antiepileptic
		Siponimod	Multiple sclerosis
		Warfarin	Anticoagulant
	CYP2B6	Efavirenz	HIV
	DPYD	Fluorouracil, Capecitabine	Cancer
	NUDT15	Azathioprine	Immunosuppressant
		Mercaptopurine, Thioguanine	Cancer
Phase II enzymes			
	UGT1A1	Irinotecan, Belinostat	Cancer
	TPMT	Azathioprine	Immunosuppressant
		Mercaptopurine, Thioguanine	Cancer
Transporters	SLCO1B1	Rosuvastatin, Simvastatin	Dyslipidemia

**Table 2:** ADME gene–drug pairs with overlap of FDA pharmacogenomic labeling (informative, actionable, testing recommended, and testing required) and CPIC evidence level.

	CPIC Level A	CPIC Level B	CPIC Level C	CPIC Level D
Informative (10)	CYP2C19-lansoprazole	CYP2D6-risperidone	CYP2D6-fluoxetine	
	CYP2D6-ondansetron		CYP2D6-galantamine	
	CYP2D6-paroxetine		CYP2D6-palonosetron	
	SLCO1B1-simvastatin		CYP2D6-quinidine	
			CYP2D6-terbinafine	
Actionable (49)	CYP2D6-amitriptyline	CYP2D6-aripiprazole	CYP2D6-darifenacin	CYB5R1-metoclopramide
	CYP2D6- atomoxetine	CYP2C19-brivaracetam	CYP2D6-duloxetine	CYB5R2-metoclopramide
	DPYD-capecitabine	CYP2D6-clomipramine	CYP2C19-esomeprazole	CYB5R3-metoclopramide
	CYP2C9-celecoxib	CYP2D6-desipramine	CYP2D6-fesoterodine	CYB5R4-metoclopramide
	CYP2C19-citalopram	CYP2C19-dexlansoprazole	CYP2C19-flibanserin	
	CYP2C19-clopidogrel	CYP2C19-doxepin	CYP2C9-flibanserin	
	CYP2D6-codeine	CYP2D6-doxepin	CYP2D6-flibanserin	
	CYP2B6-efavirenz	CYP2D6-fluvoxamine	CYP2D6-quinine	
	CYP2C19-escitalopram	CYP2D6-imipramine	CYP2C19-rabeprazole	
	DPYD-fluorouracil	CYP2D6-trimipramine	SLCO1B1-rosuvastatin	
	CYP2C9-flurbiprofen	UGT1A1-belinostat	CYP2D6-tolterodine	
	CYP2C9-meloxicam			
	CYP2D6-nortriptyline			
	CYP2C19-omeprazole			
	CYP2C19-pantoprazole			
	CYP2C9-phenytoin			
	CYP2C9-piroxicam			
	CYP2D6-pitolisant			
	CYP2D6-tamoxifen			
	CYP2D6-tramadol			
	CYP2C19-voriconazole			
	CYP2C9-warfarin			
	UGT1A1-irinotecan			
Testing Recommended (6)	NUDT15-azathioprine			
	NUDT15-mercaptopurine			
	NUDT15-thioguanine			
	TPMT-azathioprine			
	TPMT-mercaptopurine			
	TPMT-thioguanine			
Testing Required (1)	CYP2C9-siponimod			

## Data Availability

Data are contained within the article.
